# Inherited Chromosomally Integrated Human Herpesvirus 6 Demonstrates Tissue-Specific RNA Expression *In Vivo* That Correlates with an Increased Antibody Immune Response

**DOI:** 10.1128/JVI.01418-19

**Published:** 2019-12-12

**Authors:** Vikas Peddu, Isabelle Dubuc, Annie Gravel, Hong Xie, Meei-Li Huang, Dan Tenenbaum, Keith R. Jerome, Jean-Claude Tardif, Marie-Pierre Dubé, Louis Flamand, Alexander L. Greninger

**Affiliations:** a Department of Laboratory Medicine, University of Washington, Seattle, Washington, USA; b CHU de Quebec Research Center-Université Laval, Quebec City, Québec, Canada; c Montreal Heart Institute, Montréal, Québec, Canada; d Faculté de Médecine, Departement de Médecine, Université de Montréal, Montréal, Québec, Canada; e Department of Microbiology, Infectious Disease and Immunology, Faculty of Medicine, Laval University, Quebec City, Québec, Canada; f Fred Hutchinson Cancer Research Center, Seattle, Washington, USA; University of Southern California

**Keywords:** Human herpesvirus 6, iciHHV-6, ciHHV-6, HHV-6, GTEx, Genotype Tissue Expression project, LIPS, antibody response, IE1, U90

## Abstract

Human herpesviruses 6A and 6B (HHV-6A and HHV-6B) are human viruses capable of chromosomal integration. Approximately 1% of the human population carries one copy of HHV-6A/B integrated into every cell in their body, referred to as inherited chromosomally integrated human herpesvirus 6A/B (iciHHV-6A/B). Whether iciHHV-6A/B is transcriptionally active *in vivo* and how it shapes the immunological response are still unclear. In this study, we screened DNA sequencing (DNA-seq) and transcriptome sequencing (RNA-seq) data for 650 individuals available through the Genotype-Tissue Expression (GTEx) project and identified 2 iciHHV-6A- and 4 iciHHV-6B-positive candidates. When corresponding tissue-specific gene expression signatures were analyzed, low levels HHV-6A/B gene expression was found across multiple tissues, with the highest levels of gene expression in the brain (specifically for HHV-6A), testis, esophagus, and adrenal gland. U90 and U100 were the most highly expressed HHV-6 genes in both iciHHV-6A- and iciHHV-6B-positive individuals. To assess whether tissue-specific gene expression from iciHHV-6A/B influences the immune response, a cohort of 15,498 subjects was screened and 85 iciHHV-6A/B^+^ subjects were identified. Plasma samples from iciHHV-6A/B^+^ and age- and sex-matched controls were analyzed for antibodies to control antigens (cytomegalovirus [CMV], Epstein-Barr virus [EBV], and influenza virus [FLU]) or HHV-6A/B antigens. Our results indicate that iciHHV-6A/B^+^ subjects have significantly more antibodies against the U90 gene product (IE1) than do non-iciHHV-6-positive individuals. Antibody responses against EBV and FLU antigens or HHV-6A/B gene products either not expressed or expressed at low levels, such as U47, U57, and U72, were identical between controls and iciHHV-6A/B^+^ subjects. CMV-seropositive individuals with iciHHV-6A/B^+^ have more antibodies against CMV pp150 than do CMV-seropositive controls. These results argue that spontaneous gene expression from integrated HHV-6A/B leads to an increase in antigenic burden that translates into a more robust HHV-6A/B-specific antibody response.

**IMPORTANCE** HHV-6A and -6B are human herpesviruses that have the unique property of being able to integrate into the telomeric regions of human chromosomes. Approximately 1% of the world’s population carries integrated HHV-6A/B genome in every cell of their body. Whether viral genes are transcriptionally active in these individuals is unclear. By taking advantage of a unique tissue-specific gene expression data set, we showed that the majority of tissues from iciHHV-6 individuals do not show HHV-6 gene expression. Brain and testes showed the highest tissue-specific expression of HHV-6 genes in two separate data sets. Two HHV-6 genes, U90 (immediate early 1 protein) and U100 (glycoproteins Q1 and Q2), were found to be selectively and consistently expressed across several human tissues. Expression of U90 translates into an increase in antigen-specific antibody response in iciHHV-6A/B^+^ subjects relative to controls. Future studies will be needed to determine the mechanism of gene expression, the effects of these genes on human gene transcription networks, and the pathophysiological impact of having increased viral protein expression in tissue in conjunction with increased antigen-specific antibody production.

## Abstract (precis)

HHV-6A and -6B are human herpesviruses that have the unique property of being able to integrate into the telomeric regions of human chromosomes. Approximately 1% of the world’s population carries integrated HHV-6A/B genome in every cell of their body. Whether viral genes are transcriptionally active in these individuals is unclear. By taking advantage of a unique tissue-specific gene expression data set, we showed that the majority of tissues from iciHHV-6 individuals do not show HHV-6 gene expression. Brain and testes showed the highest tissue-specific expression of HHV-6 genes in two separate data sets. Two HHV-6 genes, U90 (immediate early 1 protein) and U100 (glycoproteins Q1 and Q2), were found to be selectively and consistently expressed across several human tissues. Expression of U90 translates into an increase in antigen-specific antibody response in iciHHV-6A/B^+^ subjects relative to controls. Future studies will be needed to determine the mechanism of gene expression, the effects of these genes on human gene transcription networks, and the pathophysiological impact of having increased viral protein expression in tissue in conjunction with increased antigen-specific antibody production.

## INTRODUCTION

Human herpesvirus 6 (HHV-6) represents two unique species: HHV-6A and HHV-6B. Primary HHV-6B infection occurs in 90% of children within their first 2 years of life and causes roseola, also known as sixth disease, and it has been strongly associated with febrile illness (1). HHV-6B reactivation has been observed in 56% of post hematopoietic stem cell transplant recipients. Those with posttransplant HHV-6B reactivation have also been observed to have a higher chance of human cytomegalovirus (CMV) reactivation (2).

As with all herpesviruses, HHV-6A/B establish lifelong latency, though they are unique in that they are the only human herpesviruses capable of chromosomal integration. The method of integration is thought to be the result of homologous recombination between the direct repeat (DR) regions on the right end of the HHV-6 genome and the telomeric regions of the human genome (3). In approximately 0.5 to 0.8% of the general population, integrated virus can be vertically passed through the germ line, resulting in one copy of the HHV-6A/B genome in every cell in the child’s body (4–6). This is referred to as inherited chromosomally integrated HHV-6A/B (iciHHV-6A/B) (7).

A sensitive droplet digital PCR (ddPCR)-based assay to detect a 1:1 ratio of HHV-6A/B to human cellular DNA has been described as a method of diagnosing iciHHV-6A/B (8). However, iciHHV-6A/B present a confounding issue for conventional DNA-based PCR diagnostic assays when diagnosing HHV-6A/B active infections because HHV-6A/B DNA is always present in the cells of iciHHV-6A/B patients. As a result, though the previously described ddPCR assay can be used to discriminate active infections from HHV-6A/B positive patients, it cannot determine HHV-6A/B active infection in the context of iciHHV-6A/B.

The biological consequences of having the entire HHV-6A/B genome in every cell have yet to be examined in detail. In the only large population study performed so far, Gravel et al. reported that iciHHV-6^+^ represents a risk factor for development of angina (6). Endo et al. also provided convincing evidence of reactivation of iciHHV-6A in a boy with SCID (9). Reactivation was associated with pathogenesis and was successfully treated with antivirals. At the molecular level, the left direct repeat region of the genome is fused to the telomeric region (10–12). At the other side of the genome, the right direct repeat ends with telomeric DNA repeat extensions whose lengths are often shorter than those of the other chromosomes (11). Integration in chromosome 17p is commonly seen in Europe and America, while integration in chromosome 22q is commonly observed in Asia (3, 13). Such overrepresentation in chromosomes 17p and 22q is unlikely due to multiple independent integration events but likely originates in ancestral integration events that were propagated over time (3, 13).

At present, it is unclear whether integrated HHV-6A/B genomes are transcriptionally active and, if so, whether they exhibit tissue-specific expression. In a recent study, Saviola et al. reported that the viral genome resides in a condensed nucleosome-associated state with modest enrichment for repressive histone marks H3K9me3/H3K27me3 and does not possess the active histone modifications H3K27ac/H3K4me3 (14). Whether such an epigenetic signature varies depending on the cell type and whether it is modified by *in vitro* passaging of iciHHV-6A/B^+^ subject cells remain to be determined. To study HHV-6A/B gene expression under *in vivo* conditions, we utilized the Genotype-Tissue Expression project (GTEx), which at the time of analysis contained 650 whole-blood DNA sequencing (DNA-seq) samples, to screen for iciHHV-6A/B-positive individuals. Each of the 650 DNA-Seq samples corresponds to donor identifier (ID)-containing transcriptome sequencing (RNA-seq) data for various tissues within that donor. Here we report the results of the RNA-seq screen and tissue-based iciHHV-6A/B activity from two unique gene expression data sets. Furthermore, we studied whether the HHV-6A/B gene expression detected was correlated with antigen specific antibody responses. Our hypothesis was that iciHHV-6A/B^+^ subjects may be routinely exposed to a higher antigenic burden than iciHHV-6-negative subjects and this would translate into a more robust anti-HHV-6A/B immune response.

(This article was submitted to an online preprint archive [15].)

## RESULTS

### Screening for HHV-6 in GTEx DNA-seq data reveals 6 iciHHV-6 cases among 650 individuals.

**FIG 1:**
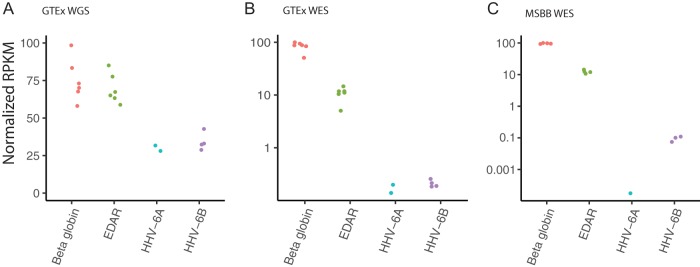
Detection of iciHHV-6A/B individuals in whole-genome sequencing (WGS) and whole-exome sequencing (WES) data from GTEx and MSBB data sets. (A) Six of 650 GTEx samples had high levels of HHV-6A/B in WGS data, consistent with iciHHV-6A/B. Normalized depth of HHV-6 compared to control gene reads (EDAR and beta-globin) yielded a ratio of 0.45 ± 0.035, consistent with one copy of iciHHV-6A/B per diploid human genome. (B) HHV-6A/B was uniquely detected in off-target reads from the same six individuals’ WES, albeit at far lower levels, consistent with the presence of off-target reads. (C) Analysis of the Mount Sinai Brain Bank WES data revealed that 4 of 350 individuals were likely positive for iciHHV-6A/B.

From the whole-genome DNA-seq data available from 650 GTEx individuals, we determined 6 cases consistent with iciHHV-6: 4 iciHHV-6B and 2 iciHHV-6A. These 6 samples had an average normalized depth of coverage across the HHV-6A/B genome that was approximately half (0.45 ± 0.035) that of human EDAR and beta-globin housekeeping genes, consistent with heterozygous iciHHV-6 at the approximate 0.8 to 1.0% prevalence typically found in the United Kingdom and United States (Fig. 1A) (4, 5). Of note, no evidence of chromosomally integrated HHV-7 was found (16).

### Off-target HHV-6 read coverage from whole-exome sequencing also correctly detects iciHHV-6A/B.

Because of the unique availability of whole-genome sequencing (WGS) and whole-exome sequencing (WES) data for most participants in the GTEx study, we examined whether WES data could be used to detect iciHHV-6A/B individuals given the comparatively large amounts of WES data available compared to WGS data. Within the GTEx WES data set, the only samples with reads aligning to HHV-6A/B were the six iciHHV-6A/B samples positive by WGS (Fig. 1B). The mean depth of coverage for HHV-6A/B in the exome data from iciHHV-6A/B individuals was 0.27×, compared to 117× for beta-globin and 14.5× for EDAR. Screening of the other 603 available whole exome sequences revealed no HHV-6A/B sequence outside the repeat regions. We used the exome screening approach to screen the Mount Sinai Brain Bank (MSBB) data set of 350 individuals and found 4 iciHHV-6 positive individuals (1 iciHHV-6A and 3 iciHHV-6B), again consistent with the expected population incidence of iciHHV-6A/B (Fig. 1C).

### Brain HHV-6 RNA expression is higher in iciHHV-6A-positive individuals than in iciHHV-6B-positive individuals, and glycoprotein U100 and IE1 U90 genes are the most highly expressed HHV-6 genes across tissues.

**FIG 2:**
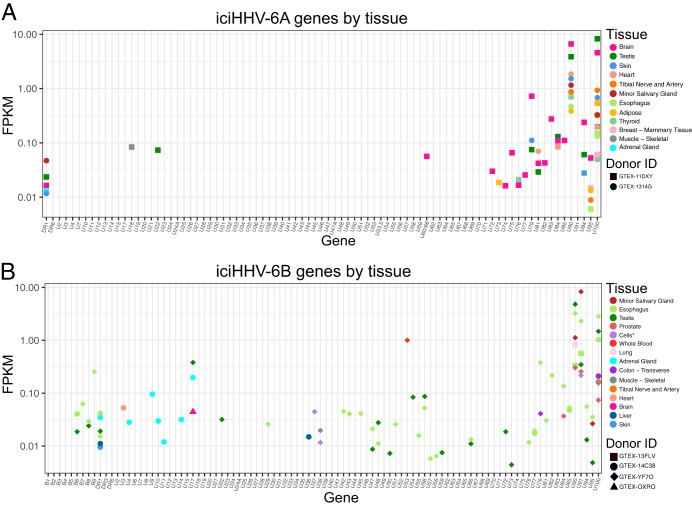
RNA-seq reads from iciHHV-6A-positive (A) and iciHHV-6B-positive (B) individuals from the GTEx data set. The highest levels of HHV-6A/B gene expression were seen in the U90 and U100 genes and in the brain (uniquely for iciHHV-6A), testis, and esophagus. The asterisk indicates that cells are transformed fibroblasts and EBV-transformed lymphocytes. FPKM, fragments per kilobase per million.

**FIG 3:**
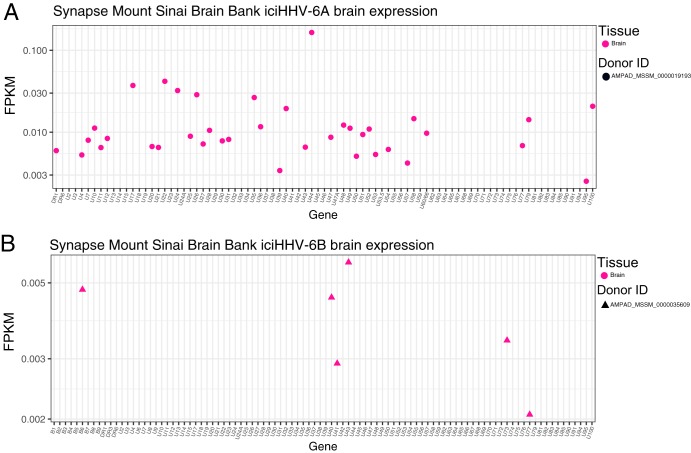
RNA-seq reads from iciHHV-6A-positive (A) and iciHHB-6B-positive (B) individuals from the Mount Sinai Brain Bank.

From the six individuals who tested positive for iciHHV-6A/B based on WGS and WES data, RNA-seq data were available from a total of 111 tissues. Analysis of these transcriptomes showed variable tissue-specific activity, with highest gene expression levels in the U90 and U100 genes for both iciHHV-6A and iciHHV-6B (Fig. 2; see also Fig. S1 to S3 and Tables S1 and S2; nucleotides 133146 to 136166 and 146696 to 150336 in the sequence with GenBank accession number NC_001664 and 134769 to 138436 and 148659 to 152335 in the sequence with GenBank accession number NC_000898). The IE1/U90 protein is among the most divergent proteins between HHV-6A and HHV-6B, with 62% identity. IE1A/U90 and IE1B/U90 are large (150 kDa) proteins involved in preventing type I interferon synthesis and signaling (17, 18). Glycoproteins Q/U100 (Q1 and Q2) are part of a multiprotein assembly responsible for receptor binding and viral entry (19, 20). iciHHV-6A expression was noticeably higher in the brain in both the GTEx and MSBB data sets than iciHHV-6B expression (Fig. 2 and 3). Viral genes were also actively expressed in the testis and esophagus for both iciHHV-6A- and iciHHV-6B-positive individuals.

### The presence of reads spanning exons, comparison of single nucleotide polymorphisms (SNPs), and insert mean sizes suggest that RNA-seq reads are from RNA.

**FIG 4:**
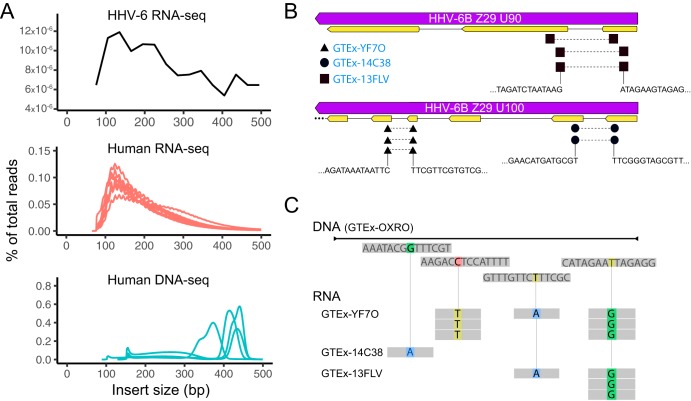
Quality control of RNA-seq reads from iciHHV-6B individuals. (A) The insert size distributions for iciHHV-6B RNA-seq, human RNA-seq, and human WGS data were compared to confirm that reads aligning to HHV-6 RNA were not due to HHV-6 DNA contamination rather than RNA. (B) Splicing in U90 and U100 was detected in the three iciHHV-6B individuals with significant RNA-seq reads. (C) Sequence polymorphisms in iciHHV-6B RNA-seq reads could also be detected in the three samples that discriminated them from each other and matched their respective consensus iciHHV-6B sequences from WGS data.

Because of the low levels of RNA expression of HHV-6A/B compared to high number of reads to HHV-6A/B in WGS data from iciHHV-6A/B-positive individuals combined with the overall perils of data mining, we performed specific quality control to ensure that preanalytical issues did not compromise our analysis. Specifically, we examined RNA-seq data for fragment insert size, splicing, and presence of unique nucleotide polymorphisms. RNA-seq reads that matched to HHV-6B from iciHHV-6B-positive individuals demonstrated the same fragment insert size as human RNA-seq reads and were noticeably different from the high insert sizes achieved for the human WGS data (Fig. 4A). We also detected reads consistent with splicing in the U90 and U100 genes for three of the four iciHHV-6B-positive individuals (Fig. 4B). Unique sequence polymorphisms in the HHV-6 RNA-seq data could be found that segregated the four iciHHV-6B sequences, and these polymorphisms matched the respective iciHHV-6B DNA sequence for that individual (Fig. 4C).

### Phylogeny reveals genetic high genetic similarity among iciHHV-6 sequences.

**FIG 5:**
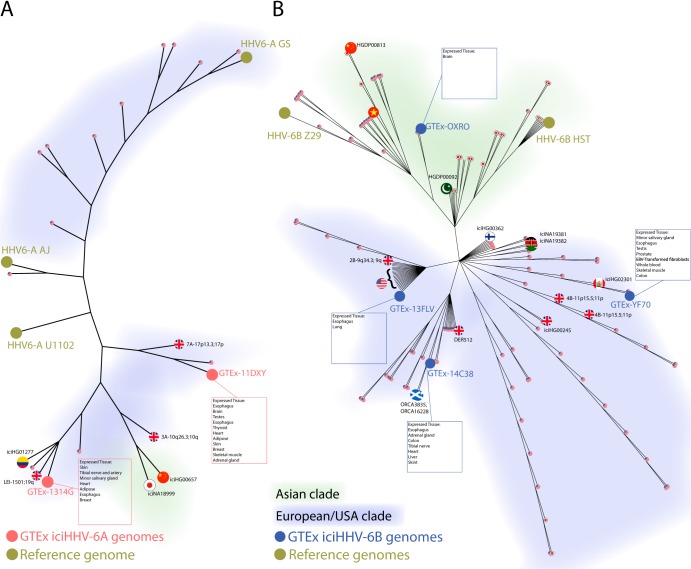
iciHHV-6A (A) and iciHHV-6B (B) sequences found in the GTEx data set cluster with previously deposited GenBank iciHHV-6 sequences. Country of origin is illustrated by country flag, while tissue RNA expression is highlighted for GTEx samples.

Phylogenetic trees reveal clustering with previously deposited iciHHV-6 sequences (Fig. 5). From the available GenBank HHV-6B sequences, two distinct HHV-6B clades are visible: one Asian and one American/European, with GTEx-OXRO falling into the former and GTEx-13LV, -14C38, and -YF70 the latter. The American clade consists of iciHHV-6B sequences from the United Kingdom and Seattle, WA, as well as HHV-6B sequences from New York (21). The Asian clade contains iciHHV-6B sequences from Pakistan and China, as well as HHV-6B sequences from Japan. It was not possible to build consensus sequences from the Synapse MSBB iciHHV-6 exome sequence samples due to insufficient coverage.

### Screening and identification of iciHHV-6^+^ subjects from the MHI Biobank.

DNA samples in the Montreal Heart Institute (MHI) Biobank from 15,498 subjects were screened by quantitative PCR (qPCR) to identify iciHHV-6A/B^+^ subjects. In total, 85 iciHHV-6A/B^+^ individuals (40 females) were identified, indicating a prevalence of 0.55% (Table 1). Of these, 46 were iciHHV-6A^+^ and 39 were iciHHV-6B^+^. All iciHHV-6A/B^+^ samples except two were confirmed by ddPCR as having 1 integrated copy of HHV-6A/B/cell. Two iciHHV-6A^+^ individuals had 2 copies. DR analysis indicated the presence of three DR copies, consistent with two adjacent viral genomes separated by one DR and flanked with one DR at each extremity. The demographics of the MHI biobank participants are provided in Table 1. Plasma samples from these 85 iciHHV-6A/B^+^ subjects and 20 controls matched for age and sex were used for the serological assay. Blood samples from 55 iciHHV-6A/B^+^ subjects and 57 controls matched for age and sex were used for plasma cytokine analyses.

**TABLE 1:** Demographics of the iciHHV-6A/B cohort and control subjects

Demographic	MHI BioBank sample (*n* = 15,498)		Subset of matched samples (*n* = 112)	
	iciHHV6 positive (*n* = 85)	iciHHV6 negative (*n* = 15,413)	iciHHV6 positive (*n* = 55)	iciHHV6 negative (*n* = 57)
Demographics				
Age (yrs), mean ± SD	62.69 ± 9.61	63.04 ± 11.42	61.98 ± 9.32	63.43 ± 9.67
Female, no. (%)	40 (47.05)	6,400 (41.52)	27 (49.09)	27 (47.37)
Race, no. (%)				
Asian	0	58 (0.38)		
Black	0	113 (0.73)		
Caucasian	84 (98.82)	15,102 (97.98)	55 (100.0)	54 (94.74)
Hispanic	1 (1.18)	110 (0.71)		
Native American	0	25 (0.16)	0	3 (5.26)
Other	0	2 (0.01)		
HHV6 type, no. (%)				
A	46 (54.12)		28 (50.91)	
B	39 (45.88)		27 (49.09)	

### Plasma cytokine analysis.

A summary of plasma cytokine results is presented in Table S3. Of the 14 analytes measured, only tumor necrosis factor alpha (TNF-α) showed a statistically significant difference between controls and iciHHV-6A^+^ subjects (*P* = 0.02). All other cytokine levels were comparable between iciHHV-6A^+^ subjects, iciHHV-6B^+^ subjects, and controls.

### Antibody response of iciHHV-6A/B^+^ individuals against control antigens.

**FIG 6:**
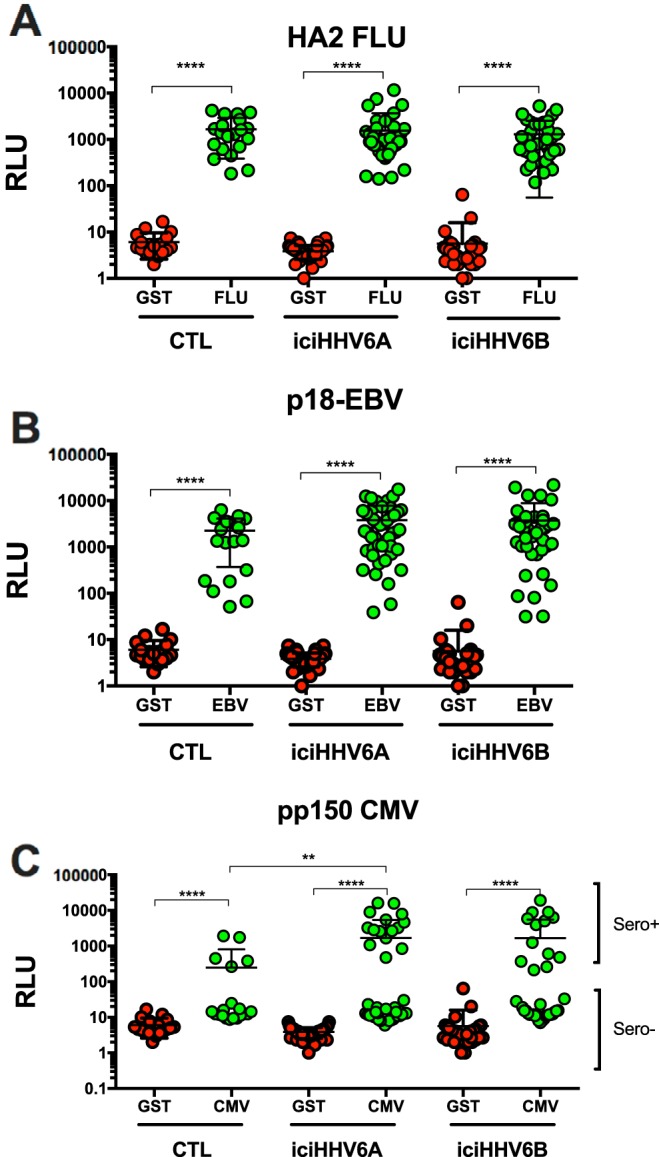
Detection of antibodies against control antigens from influenza A virus (A), Epstein-Barr virus (B), and cytomegalovirus (C) using LIPS. Lysates containing antigens were incubated with sera from control (*n* = 20), iciHHV-6A^+^ (*n* = 46), and iciHHV-6B^+^ (*n* = 38) subjects to determine reactivity. Each dot represents the mean ± SD of duplicate assays for each donor. ****, *P* < 0.0001; **, *P* < 0.01.

The sera of 46 iciHHV-6A^+^ individuals, 38 iciHHV-6B^+^ individuals, and 20 controls matched for age and sex were analyzed for reactivity against influenza (FLU; hemagglutinin [HA]), Epstein-Barr virus (EBV; p18), and cytomegalovirus (CMV; pp150) antigens. The luciferase–glutathione S-transferase (GST) fusion antigen was used as a negative control. Expression of the fusion proteins was determined by Western blotting (Fig. S4). Relative to the negative-control antigen, all sera showed >2 log_10_ reactivity against the FLU antigen, with no significant differences between the groups (Fig. 6A). Similar results were obtained with the EBV antigen with the exception that iciHHV-6A^+^ and iciHHV-6B^+^ subjects had slightly higher (1.7×) antibody levels than control subjects (Fig. 6B). Such a difference did not, however, reach statistical significance. Results for the CMV pp150 antigen indicate that the cohort contains both CMV-seronegative and -seropositive subjects (Fig. 6C). The proportion of seropositive subjects varied between 25 and 30% and was not different between groups. Intriguingly, among CMV-seropositive subjects, iciHHV-6A^+^ and iciHHV-6B^+^ subjects displayed >5× higher antibody reactivity against the CMV antigen than control subjects, with such a difference reaching statistical significance for the iciHHV-6A^+^ group (*P* < 0.01).

### Antibody response of iciHHV-6A/B^+^ individuals against HHV-6A/B antigens.

**FIG 7:**
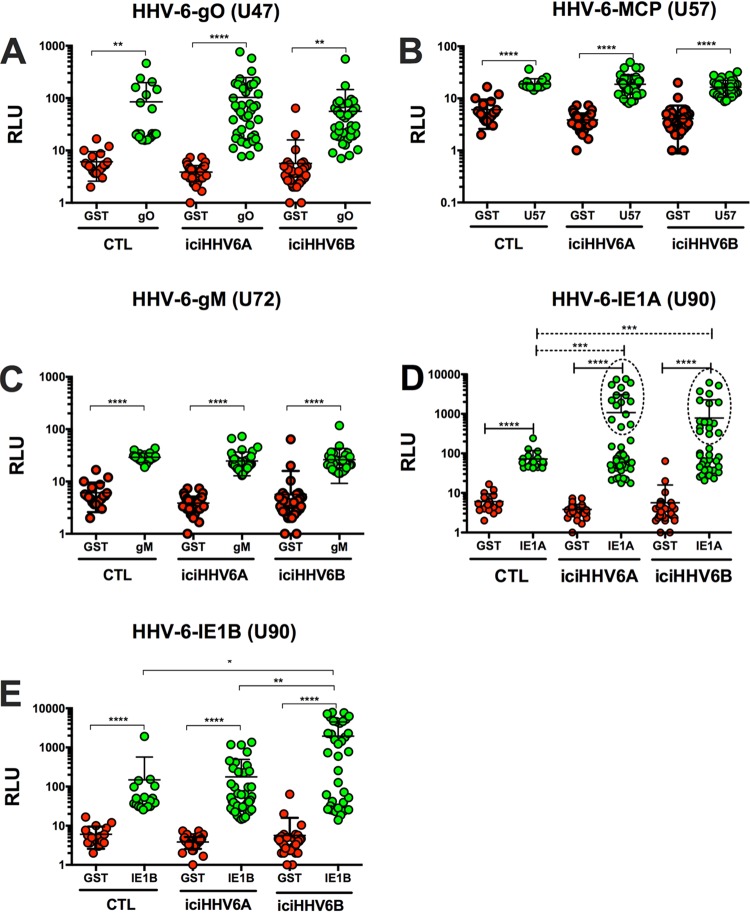
Detection of antibodies against HHV-6A/B antigens (gO/U47 [A], MCP/U57 [B], gM/U72 [C], IE1A/HHV-6A U90 [D], and IE1B/HHV-6B U90 [E]) using LIPS. Lysates containing antigens were incubated with sera from control (*n* = 20), iciHHV-6A^+^ (*n* = 46), and iciHHV-6B^+^ (*n* = 38) subjects to determine reactivity. For panel D, two groups of responders were identified. Those with reactivities similar to controls and those with high IE1A/U90 reactivity, identified as those with reactivities above the mean + 2 SD of controls (dashed ovals). High responders were found to have significantly higher antibodies to IE1A/U90 than controls. Each dot represents the mean ± SD of duplicate assays for each donor. ****, *P* < 0.0001;***, *P* < 0.0005; **, *P* < 0.001; *, *P* < 0.05.

The GTEx results indicate that certain HHV-6A/B genes, such as U90 (IE1) and U100 (gQ), are preferentially expressed relative to others, such as U47 (gO), U57 (MCP), or U72 (gM). Whether this would translate into a differential antibody response was examined next. Constructs expressing Ruc-gO/U47, Ruc-MCP/U57, Ruc-gM/U72, and Ruc-IE1A/U90 or Ruc-IE1B/U90 were generated and expression was verified by Western blotting (Fig. S4). Most HHV-6A/B proteins share >85% (often >95%) identity at the amino acid level, making it very difficult to discriminate whether antibodies are specific to HHV-6A or HHV-6B proteins. For this reason, reactivities against gO/U47 (87% identity), gM/U57 (97% identity), and MCP/U72 (97% identity) were measured using HHV-6B proteins. Serum reactivity against gO/U47, gM/U57, and MCP/U72 was detected in most subjects, with no significant differences observed between iciHHV-6A^+^ subjects, iciHHV-6B^+^ subjects, and control subjects (Fig. 7A to C). Considering that IE1/U90 is the most divergent protein between HHV-6A and HHV-6B (62% identity), antibody reactivities against both proteins were measured. Although higher reactivity of sera from iciHHV-6A^+^ and iciHHV-6B^+^ subjects against the IE1A/U90 antigen was recorded than for controls, the results did not reach statistical significance (*P* > 0.05) (Fig. 7D). Of interest, approximately one-third of the iciHHV-6^+^ subjects had significantly more antibodies (25×) against IE1A/U90 than controls (Fig. 7D, dashed ovals). When high responders were compared to controls, highly statistical significance was observed. Analysis of antibody response against IE1B/U90 also indicated that iciHHV-6B^+^ subjects had significantly more antibodies than control subjects or iciHHV-6A^+^ subjects (Fig. 7E). The mean antibody response against IE1B/U90 of iciHHV-6B^+^ individuals was 13× greater than that of control subjects (*P* < 0.05).

## DISCUSSION

Here we show that iciHHV-6-positive individuals show tissue-specific expression of HHV-6 genes *in vivo* and that the highly expressed U90 gene is associated with a stronger immune response in iciHHV-6A/B^+^ individuals. We detected a 0.92% prevalence of iciHHV-6A/B^+^ in the GTEx cohort, while in the MHI cohort, 0.55% of individuals were iciHHV-6A/B^+^, both matching the previously reported rate of 0.5% (22). Our analysis of the GTEx RNA-seq files revealed that iciHHV-6A and -6B are not equally active in all tissues, consistent with tissue-specific gene expression. Where HHV-6A/B gene expression could be detected, there was consistently high expression of the U90 and U100 genes relative to other genes for both iciHHV-6A and iciHHV-6B.

We also found that coverage from WES data could prove to be a reliable metric for detecting iciHHV-6A/B in WES sequence. In the GTEx data set we first analyzed WGS data to find possible iciHHV-6A/B candidates and confirmed the candidates by looking for a 1:2 ratio of coverage for housekeeping genes to HHV-6A/B genomes. Out of the 650 available WES samples in the GTEx data set, the only ones that contained sequencing reads to HHV-6A/B were the six samples confirmed by our WGS screen to be iciHHV-6A/B^+^. Given the significantly greater number of individuals with WES data than with WGS data, the ability to determine iciHHV-6A/B status via WES data alone substantially expands the numbers and types of studies of iciHHV-6A/B status that can be performed.

Within the GTEx iciHHV-6A/B-positive samples, brain tissue was observed to be relatively highly active, with 11 different viral genes expressed. We also screened the Synapse Mount Sinai Brain Bank (MSBB) data set and found three iciHHV-6B sequences and one iciHHV-6A sequence. Analysis of the corresponding RNA-seq reads revealed that HHV-6 was expressed in the frontal pole, superior temporal gyrus, parahippocampal gyrus, and interior frontal gyrus. Of the three iciHHV-6B-positive samples, only one was found to express HHV-6B genes. This sample, AMPAD_MSSM_0000035609, was active only in the parahippocampal gyrus and had only 6 genes expressed, as opposed to 38 genes expressed for the iciHHV-6A sample (Fig. 4). Values for reads per kilobase per million (RPKM) for HHV-6B RNA expression in the brain in iciHHV-6B individuals in the MSBB cohort were roughly 2 to 4 logarithms lower than in other tissues in the GTEx expression data.

Considering that iciHHV-6A/B^+^ individuals have 1 copy of the entire viral genome in every cell, it is expected that at any given time, certain viral genes might be expressed. At present it is unclear what stimulates viral gene expression from integrated HHV-6A/B. Certain genes, such as U90 and U100, are expressed more readily than others, as evidenced by the GTEx data. Of interest, recent results by Gravel et al. using *in vitro*-derived cellular clones with chromosomally integrated HHV-6A also identified U90 and U100 as genes whose expression could readily be demonstrated (23). Expression of U90 and U100 was, however, clone specific, suggesting that the chromosome carrying the integrated virus might influence viral gene expression.

The biological consequences of carrying iciHHV-6A/B^+^ remain poorly characterized. In the current work, we provide evidence that the magnitude of the antibody responses of iciHHV-6A/B^+^ subjects correlates with the expression of viral genes. The antibody responses against low-level-expressed genes such as U47, U57, and U72 are similar between controls and iciHHV-6A/B^+^ subjects. In contrast, the antibody responses of iciHHV-6A/B^+^ subjects against gene products, such as U90, which are highly expressed are much greater than those of control individuals. Whether this increase in antibody against HHV-6A/B antigens offers increased protection against HHV-6 reactivation or reinfection is presently unknown. However, considering that all cells of iciHHV-6A/B^+^ subjects have the potential to express viral proteins, these may, if appropriately presented or exposed to the cell surface, represent targets for immune attacks leading to cell destruction. Over time (decades), such chronic immune destruction may result in various pathological conditions depending on the affected tissues.

Our results also suggest that iciHHV-6A/B^+^ subjects have higher antibody levels than controls against EBV and CMV. Previous work indicated that HHV-6A promotes the reactivation of EBV (24) through activation of the EBV Zebra promoter (25). Immediate-early or early gene products were thought to be responsible for activation of the EBV Zebra promoter (25). Similarly, a recent report indicated that subjects with documented HHV-6B reactivation had a 15× increase in CMV reactivation rates, suggesting that HHV-6B may, directly or indirectly, trigger CMV reactivation (2). Reactivation of EBV and/or CMV by HHV-6A/B would therefore cause an increase viral antigenic burden, resulting in increased antibody production. Expression of viral transactivators such as U90 may be sufficient to initiate the reactivation of CMV or EBV from latently infected cells.

Since much of the work described here was secondary data analysis, our study has a number of limitations. We were unable to obtain tissues or additional metadata from either the GTEx or MSBB data sets to confirm our work. We cannot rule out the possibility of preanalytical errors such as trace DNA contamination being the cause of low levels of iciHHV-6 RNA expression. Similarly, we were unable to confirm HHV-6A/B gene or protein expression by orthogonal methods such as immunohistochemistry or reverse transcription (RT)-qPCR. Alternatively, it is difficult to rule out the possibility that viral gene expression or reactivation may occur postmortem, although all GTEx autopsies were performed within 24 h of death (26, 27). Where possible, we used specific HHV-6 SNPs to ensure that no cross-sample contamination could account for recovery of HHV-6A/B reads, and we confirmed every read by BLASTn analysis to the NCBI nucleotide database.

Despite these limitations, our work provides the first demonstration of *in vivo* expression of HHV-6 genes from integrated HHV-6A/B in various tissues (28, 29). The fact that brain tissue was the most active in transcribing iciHHV-6A genes is of potential interest considering the association of HHV-6A with various neurogenerative diseases, such as multiple sclerosis (MS) and Alzheimer’s disease (30, 31). Furthermore, these *in silico* analyses could be correlated with actual biological data from a large cohort of iciHHV-6A/B^+^ subjects. Considering the prevalence of iciHHV-6A/B (0.5 to 1%), only through analyses of very large biobanks with full medical record will the etiology of diseases linked with iciHHV-6A/B be unraveled.

## MATERIALS AND METHODS

### GTEx data analysis.

GTEx genotype data were downloaded from dbgap (1 June 2018) using prefetch. A total of 650 DNA sequence SRA files were clipped, decompressed, and extracted using fastq-dump with the following flags: –W (removes tag sequences from data set), –I (uniquely labels paired-end reads), and –split-files (splits paired-end reads into separate files). The fastq-dump output was piped to Bowtie2 (32) for alignment using the –no-unal (unaligned reads not saved in resulting SAM file) and –-local (local alignment) flags. FASTQ files were aligned to the HHV-6A U1102 (GenBank accession number NC_001664.4), HHV-6B Z29 (GenBank accession number AF157706.1), and HHV-7 RK (GenBank accession number NC_001716.2) reference genomes, with any repeat like regions manually removed.

For the 6/650 files suspected to be positive for iciHHV-6A/B (e.g., >25× average depth across HHV-6A/B reference genomes in the DNA-seq data set), corresponding RNA-seq FASTQ files for all available tissues (111 total) were downloaded and aligned to HHV-6A/B reference genomes as described above. All reads were confirmed as HHV-6A/B using BLAST with an E value of <1e−8 against the NCBI nucleotide database (5 January 2019). For corresponding negative controls, 100 GTEx biospecimen IDs were randomly selected and all available RNA-seq FASTQ files (1,903 total) corresponding to those individuals were aligned against HHV-6A/B references as described above.

DNA-seq FASTQ reads corresponding to the six iciHHV-6 positive donors were aligned to portions of the human EDAR (GenBank accession number NM_022336.4) and beta-globin (GenBank accession number AH001475.2) genes that were trimmed of human repeats as well as repeat-trimmed HHV-6A U1102 (GenBank accession number NC_001664.4) and HHV-6B Z29 (GenBank accession number AF157706.1) reference genomes, using with the same Bowtie2 options as specified above. The beta-globin and EDAR genes were chosen because they are a common human housekeeping genes used in our qPCR work. We calculated a normalized depth of coverage by counting the number of reads aligned to the region of interest (*R*) divided by the total length of the sequence in megabases (*B*) from the sample and normalizing the highest RPKM from EDAR or beta-globin obtained to 100 × [(*R* × 30,000)/*B*].

### GTEx RNA-seq quality control analyses.

To calculate fragment insert size distribution of the human WGS and human RNA-seq reads, a random subsample of 10 million reads was taken from GTEx WGS data for each of the four iciHHV-6B-positive individuals as well as the 7 RNA-seq samples that showed the highest HHV-6 gene expression. Alignment was performed to hg38 and HHV-6 reference genomes using Bowtie2 with the same flags as described above. Because of the low numbers of RNA-seq reads to HHV-6A/B, these reads were pulled from the resulting SAM files and analyzed as one distribution.

HHV-6B RNA-seq reads for each iciHHV-6B-positive individual were aligned to the GTEx-OXRO consensus genome using the Geneious read mapper and manually inspected for variants that could differentiate each donor. Alignment of paired reads to the HHV-6B Z29 reference genome using Geneious was also performed to find paired reads spanning splice junctions.

### MSBB data.

Whole-exome BAM files aligned to hg19 were downloaded (August 2018) from the Synapse Mount Sinai Brain Bank (MSBB) database (33). Unmapped reads were extracted via samtools using the command “samtools view -b -f 4 <BAM file>” and converted into FASTQ files via samtools bam2fq (34). FASTQ files were then combined and aligned to HHV-6A and HHV-6B reference genomes as described above. Twenty randomly selected samples that were negative for HHV-6 DNA were also screened for HHV-6 RNA. All reads were confirmed as HHV-6 using BLASTn, with an E value of <1e−8 against the NCBI nucleotide database (5 January 2019).

### Phylogenetic trees.

HHV-6A and HHV-6B genomes were downloaded from NCBI GenBank (1 September 2018). Contiguous HHV-6B sequences between nucleotides 9515 and 118889 of the Z29 reference sequence (GenBank accession number AF157706.1), corresponding to genes U4 to U77, were used for analysis due to a lack of missing sequence in this region. Contiguous HHV-6A sequences between nucleotides 79352 and 110248 of the U1102 reference sequence (GenBank accession number NC_001664.4) were similarly used, corresponding to genes U48 to U73. Both sets of subsequences were aligned with MAFFT using default parameters. Phylogenetic trees were constructed using the Geneious tree builder with 100 bootstrap iterations.

### Montreal Heart Institute Biobank screening.

The MHI Biobank contains DNA, buffy coats, and aliquots of plasma for every patient (*n* = 15 498). Basic demographic data of the subjects are presented in Table 1. For identification of iciHHV-6A/B^+^ subjects, screening was carried out using the Roche LightCycler 480 Instrument II real-time PCR apparatus and 384-well plates containing DNA samples from the MHI Biobank using a previously described protocol (6). Samples positive for iciHHV-6A/B were confirmed by ddPCR as previously described (8, 23). All patients provided consent, and the study was approved by the Montreal Heart Institute Ethics Committee.

### Plasma cytokine analysis.

Plasma was analyzed using the human high-sensitivity T-cell 14-plex assay from Eve Technologies (Calgary, Alberta, Canada). Analytes included granulocyte-macrophage colony-stimulating factor (GM-CSF), gamma interferon (IFN-γ), interleukin 1β (IL-1β), IL-2, IL-4, IL-5, IL-6, IL-8, IL-10, IL-12p70, IL-13, IL-17A, IL-23, and TNF-α.

### LIPS.

The luciferase immunoprecipitation assay system (LIPS) has been described previously (35, 36). In brief, genes of interest are cloned in frame with a FLAG-tagged *Renilla* luciferase (Ruc) gene using the pREN2 vector. All clones were verified by restriction enzyme profiling and DNA sequencing. The following constructs were previously described and generously provided by Peter Burbelo (NIH): pREN2-p18 EBV, pREN2-pp150-d1 CMV, and pREN2-HA2 influenza (37). The entire HHV-6B U57 gene was first cloned into pENTR-FLAG by Gibson assembly cloning. Then the BamHI/SmaI fragment containing the U57 coding region from positions 1 to 902 was subcloned into pREN2 to yield pREN2-HHV-6B MCP/U57. The entire HHV-6B gM gene (U72 amino acids [aa] 1 to 344) was cloned by Gibson assembly cloning into BamHI-Sca1-digested pREN2 to yield pREN2-HHV-6B gM/U72. The entire HHV-6B gO gene (U47 aa 1 to 738) was cloned by Gibson assembly cloning into BamHI-Sca1-digested pREN2 to yield pREN2-HHV-6B gO/U47. pREN2-IE1B/U90 (aa 1 to 857) was created by subcloning a BamHI/KpnI fragment from the pMalC2-IE1B/U90 vector into BamHI/KpnI-digested pREN2. The pREN2-IE1A/U90 (aa 24 to 941) vector was generated by subcloning a BamHI/XhoI fragment from pcDNA4TO-IE1A/U90 into BamHI/XhoI-digested pREN2 vector. The pREN2-GST negative-control vector was generated by subcloning the GST gene using the NcoI-blunted XbaI fragment obtained from pENTR4-GST 6P-1. pENTR4-GST 6P-1 (w487-1) was a gift from Eric Campeau and Paul Kaufman (Addgene; plasmid number 17741) (38). The insert was ligated in BamHI-blunted, XbaI-digested pREN2 vector. Protein expression was validated by Western blotting using anti-FLAG antibodies. To prepare lysates for the LIPS assay, HEK293T cells seeded the day before at 4 × 10^6^/10-cm^2^ petri dish were transfected with 8 μg of vectors using polyethylenimine. Forty-eight hours posttransfection, cells were harvested and lysed as previously described (35, 36). Each serum was tested in duplicate at a final dilution of 1:100. Sera were incubated with the lysate containing 10^6^ relative luciferase units (RLU) of the target antigen in wells of a 96-well plate for 2 h at room temperature with shaking (300 rpm), after which protein A-coated magnetic spheres were added to the wells with moderate shaking (300 rpm). After 60 min, the plates were loaded onto an automatic enzyme-linked immunosorbent assay (ELISA) plate washer equipped with a magnetic stand. After 3 washes, the buffer was removed, and the plates were loaded into a luminometer with an automatic substrate dispenser (M200 reader; Tecan, Morrisville, NC). Light emission was measured over 10 s with a 2-s start delay.

### Statistical analysis.

Analyses of cytokine levels were performed using nonparametric Kruskal-Wallis test followed by Dunn’s multiple-comparison test. For antibody levels, statistical significance was determined using the nonparametric Mann-Whitney test. A *P* value of <0.05 was considered significant.

## Supplementary Material

Supplemental file 1

Supplemental file 2
