# Supplementary material for: Inherited Chromosomally Integrated Human Herpesvirus 6 Demonstrates Tissue-Specific RNA Expression In Vivo That Correlates with an Increased Antibody Immune Response
Source: J Virol. 2019 Dec 12;94(1):e01418-19. doi: 10.1128/JVI.01418-19 (PMC6912112; doi:10.1128/JVI.01418-19)
Supplement: Supplemental file 2 [file JVI.01418-19-s0002.pdf]

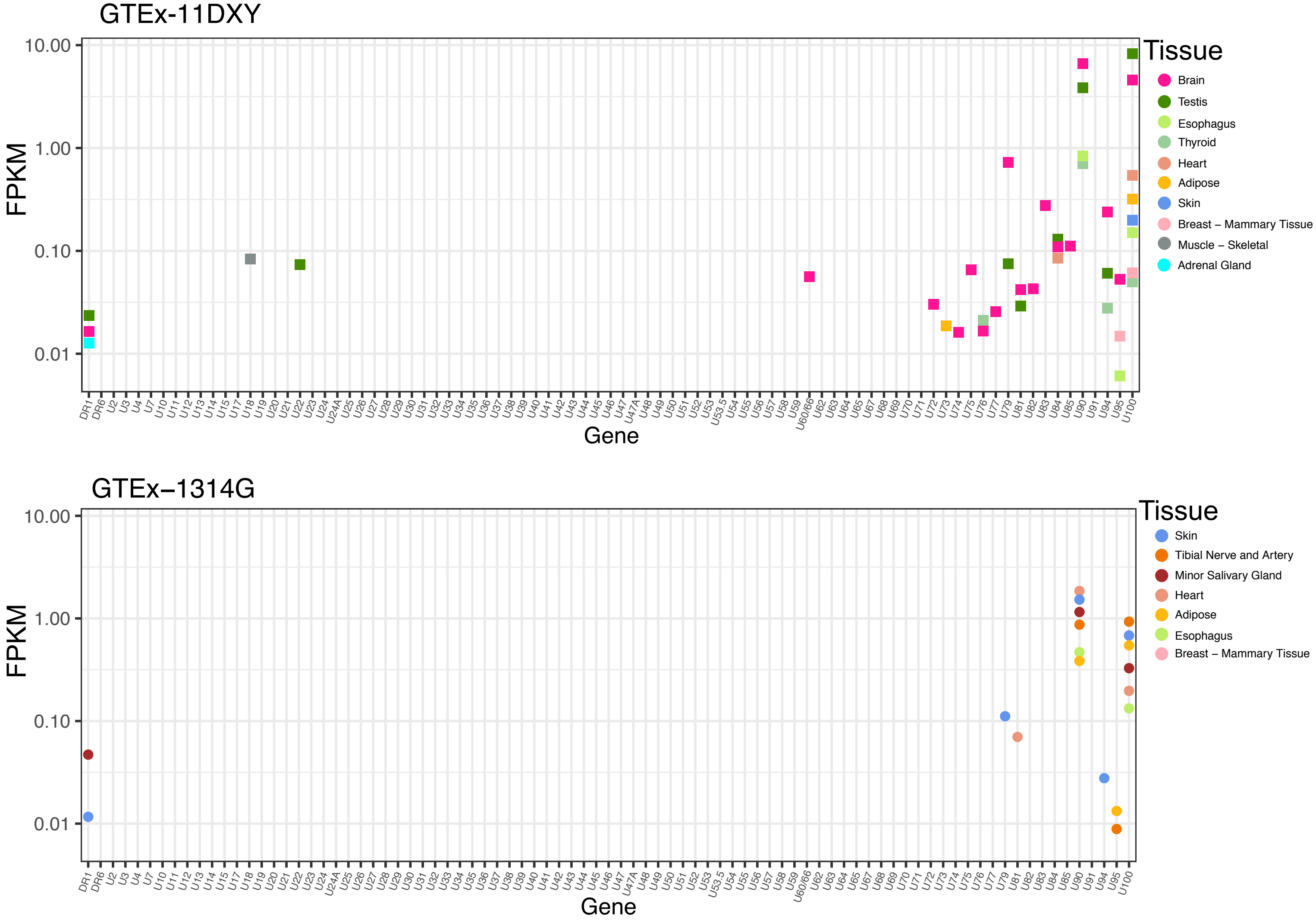

Supplemental Figure 1 – Individual level iciHHV-6A gene expression data from the two iciHHV-6A positive individuals from GTEx.

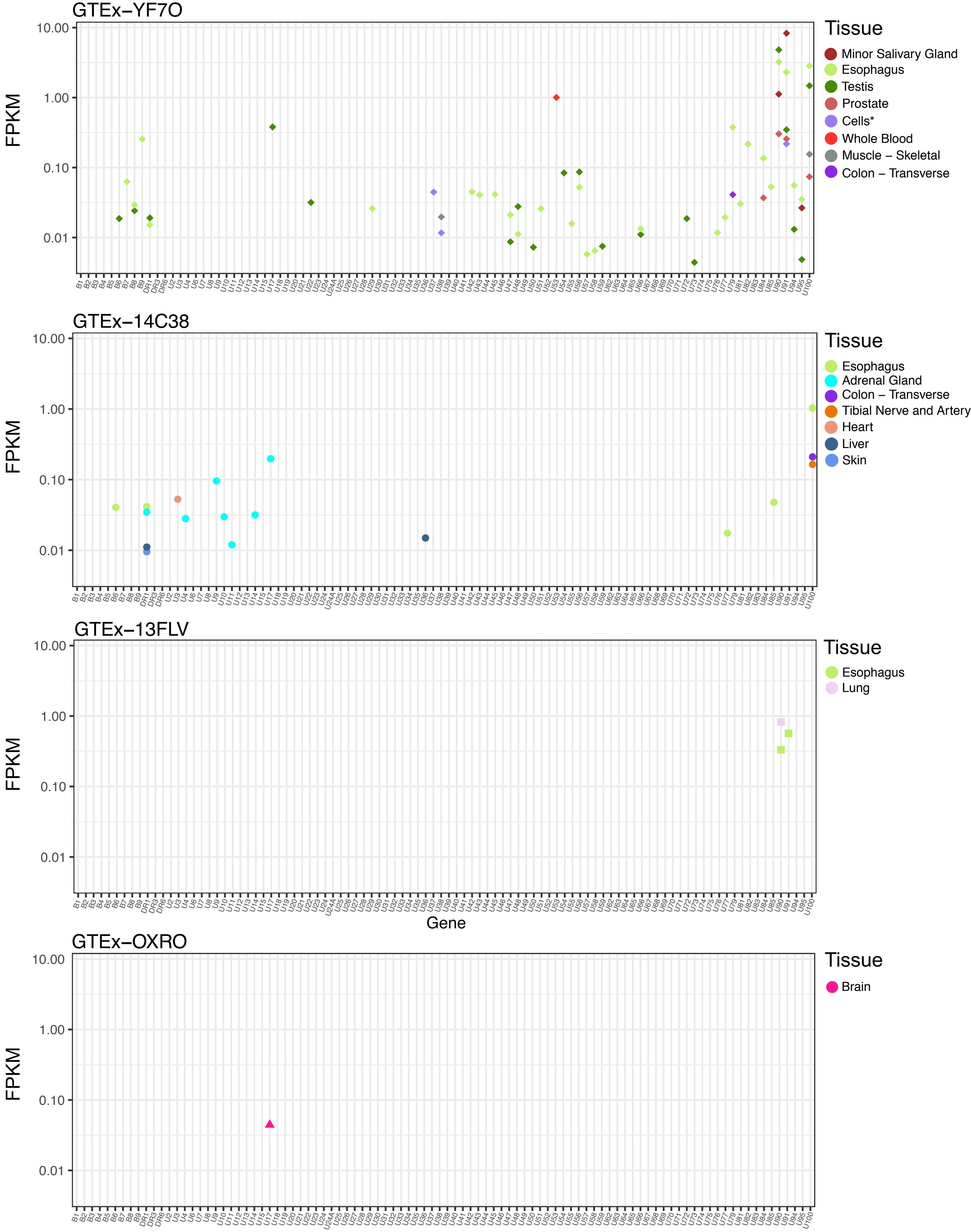

Supplemental Figure 2 – Individual level iciHHV-6B gene expression data from the four iciHHV-6B positive individuals from GTEx.

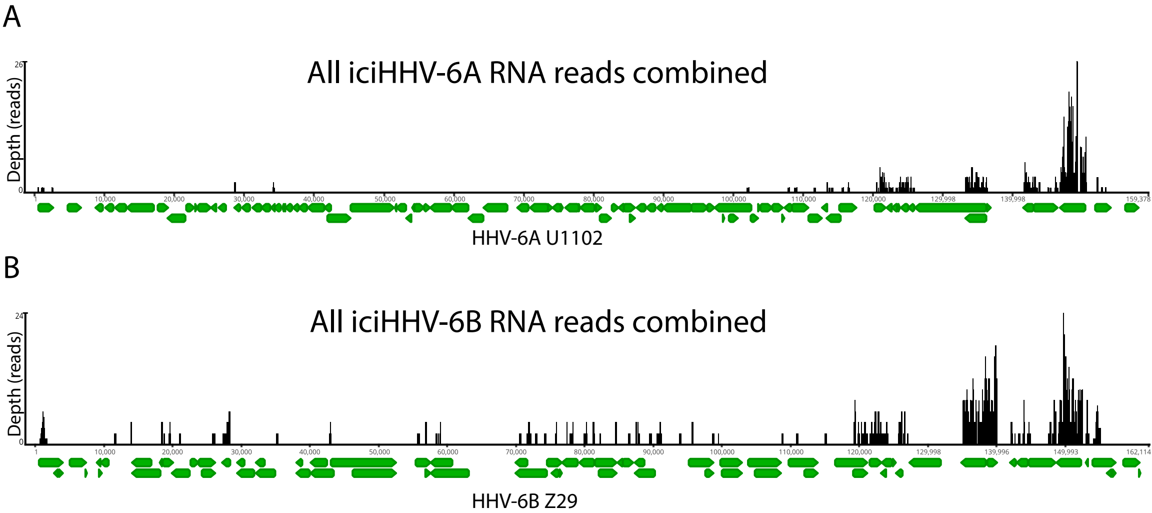

Supplemental Figure 3 – Overall depth of all HHV-6A (A) and HHV-6B (B) RNA-Seq reads across all tissues, demonstrating highest coverage of the U90 and U100 genes.

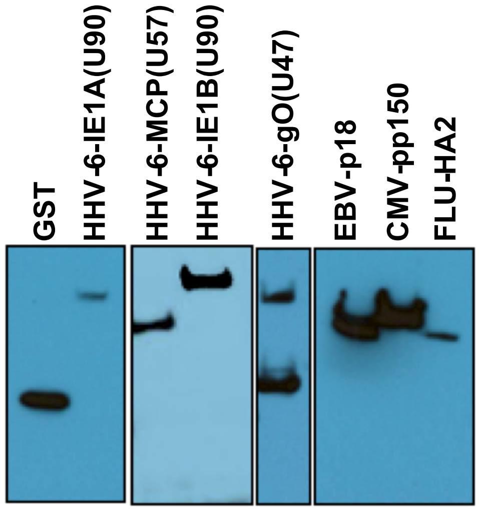

Supplemental Figure 4 - Expression and detection of antigens used for LIPS assay. Vectors expressing control (GST, HA-FLU, p18-EBV, pp150-CMV) or HHV-6 antigens (gO/U47, MCP/U57, IE1A/U90, IE1B/U90) were transfected in HEK293T cells. Forty-eight hours later, expression of proteins was assessed by western blot using anti-FLAG antibodies.
